# Impact of a Smoking Cessation Quitline in Vietnam: Evidence Base and Future Directions

**DOI:** 10.3390/ijerph16142538

**Published:** 2019-07-16

**Authors:** Chau Quy Ngo, Phuong Thu Phan, Giap Van Vu, Quyen Thi Le Pham, Hanh Thi Chu, Kiet Tuan Huy Pham, Bach Xuan Tran, Huyen Phuc Do, Cuong Tat Nguyen, Tung Thanh Tran, Giang Hai Ha, Anh Kim Dang, Huong Thi Lan Nguyen, Carl A. Latkin, Cyrus S.H. Ho, Roger C.M. Ho

**Affiliations:** 1 Respiratory Center, Bach Mai Hospital, Hanoi 100000, Vietnam; 2 Department of Internal Medicine, Hanoi Medical University, Hanoi 100000, Vietnam; 3 Institute for Preventive Medicine and Public Health, Hanoi Medical University, Hanoi 100000, Vietnam; 4 Department of Health, Behavior and Society, Johns Hopkins Bloomberg School of Public Health, Baltimore, MD 21205, USA; 5 Center of Excellence in Health Services and System Research, Nguyen Tat Thanh University, Ho Chi Minh City 700000, Vietnam; 6 Institute for Global Health Innovations, Duy Tan University, Da Nang 550000, Vietnam; 7 Center of Excellence in Evidence-based Medicine, Nguyen Tat Thanh University, Ho Chi Minh City 700000, Vietnam; 8 Vietnam Department of Psychological Medicine, National University Hospital, Singapore 119074, Singapore; 9 Center of Excellence in Behavioral Medicine, Nguyen Tat Thanh University, Ho Chi Minh City 700000, Vietnam; 10 Department of Psychological Medicine, Yong Loo Lin School of Medicine, National University of Singapore, Singapore 119077, Singapore; 11 Institute for Health Innovation and Technology (iHealthtech), National University of Singapore, Singapore 119077, Singapore

**Keywords:** smoking, quitting, nicotine dependence, quitline, Vietnam, smoking behaviors, impact

## Abstract

Smoking is considered the most critical modifiable factor with regard to lung cancer and remains a public health concern in many countries, including Vietnam, which is among those countries with the highest tobacco consumption rates in the world. This study has examined the impact of national telephone counselling for smoking cessation and has identified the factors associated with the impact of the quitline among male callers in Vietnam. A randomized cross-sectional survey of 469 smokers who sought smoking cessation services via the national quitline was performed from September 2015 to May 2016. The primary outcomes were measured by a self-reported quit rate at the time of assessment, 7 day point prevalence abstinence (PA), 6 month prolonged PA, service satisfaction, and level of motivation. Among the participants, 31.6% were abstinent, and 5.1% of participants successfully stopped smoking and did not need to seek quitline support. Most of the clients were satisfied with the quality of service (88.5%), felt more confident about quitting (74.3%), and took early action via their first quit attempt (81.7%); 18.3% reported a more than 7 day abstinence period at the time of survey. The primary reasons for smoking relapse were surrounding smoking environments (51.6%) and craving symptoms (44.1%). Future smoking cessation efforts should focus on improving the quality of quitline services, client satisfaction, and developing a tailored program and counseling targeting smokers with specific characteristics, especially ones experiencing chronic diseases.

## 1. Introduction

Smoking is a modifiable leading cause of mortality and morbidity. Smoking is responsible for 5% of worldwide deaths, and smoking-related diseases are attributed to 14% of deaths among adults aged from 30 years of age or older [1]. Smoking-related mortality accounts for 6.2% of total deaths [2]. Additionally, tobacco consumption, especially in early age (from 15 to 59), threatens 61% of the workforce globally due to the high risk of smoking-related illnesses [3], low productivity among workers in smoke environments [4,5] and tobacco-related healthcare costs [6]. Thus, smoking cessation is highly recommended because quitting smoking results in substantial health improvements, which are measurable immediately after 20 min of the last cigarette [7,8].

Vietnam is experiencing rapid epidemiologic and demographic shifts. Chronic diseases now account for 75% of the total disease burden [9] and tobacco use is one of the leading preventable causes, with 45.3% of males smoking 15.6 million adults) [10]. It has been estimated that all smokers paid 31,000 billion VND (USD 1409 million) to buy cigarettes in 2015 rather than investing in education, health care, or business [11]. As consequence, Vietnam is struggling with 24,679.9 billion Vietnamese dong (VND) (equivalent to USD 1173.2 million) of the total costs related to smoking, which are responsible for approximately 1% of the 2011 national gross domestic product [12]. It has been estimated that the direct costs of healthcare related to smoking are 12,463.4 billion VND (USD 592.4 million) [13]. Notwithstanding, smoking cessation remains under-emphasized in Vietnam, especially in rural areas. Despite 53.6% (approximately 8.3 million) of current smokers intending to quit [11], they do not have too much choice in quitting because nicotine replacement therapy (NRT) is not legally sold and smoking cessation support and treatment are only available in some hospitals [10]. The healthcare system in Vietnam is covered from the grassroots with commune health centers and many village health workers. However, 53.2% of these workers have been found not to have the skills to provide smoking cessation counseling for their patients [14]. Thus, smokers who wish to quit have to mainly use self-help as the most common and affordable method of smoking cessation.

Vietnam has enforced the 2013 Law on Prevention of Tobacco Harms, which targets the development and operation of multi-sectoral policies towards smoke-free environments and reducing the prevalence of non-communicable diseases [9,15]. Thus, having been launched in 2015, the national counseling service quitline has been expected to provide toll-free telephone-based counseling with nationally standardized, skillful, and professional counselors for seekers who intend to cease smoking and resist relapse. Vietnamese smokers may find useful information about the national quitline via national anti-smoking education and communication programs in mass media (e.g., TV advertisements, social networks, and health providers). If interested, they can call the quitline and register to participate in counselling for quit plans. Although there are many types of smoking cessation interventions, free-of-charge telephone counselling with a skillful counselor available to talk about quit plans with callers is considered a cost-effective approach to reduce the prevalence of current smokers [16] and improve self-efficacy and motivation to reach prolonged abstinence [17].

The efficacy of telephone counseling for smoking cessation has been well-documented worldwide: it helps to increase the quit rate from 7% to 10%, according to a Cochrane review of 104 studies [18]. However, very little is known in developing countries like Vietnam. Cessation is required for better health, but to our knowledge, there has been no published research about the impact of smoking cessation using the quitline in Vietnam, and there is still a paucity of evidence on the outcomes and future directions of the operation of the quitline after five years of operation. Our previous study has found some factors associated with quitting attempts among callers of the quitline. We found that 75.9% of callers intended to quit smoking, but 90.8% of them were not confident enough to take action [17]. Thus, this present study aims to evaluate the impact of the national quitline’s smoking cessation services on the change of smoking behaviors and identify the associated factors that influence quitline outcomes in Vietnam. Results from this study will advance our knowledge on how a smoking cessation based on telephoning approach can help to reduce the prevalence of current smokers and improve wellbeing for both callers and their family. This evidence will also contribute to providing important implications for improving the service quality of the quitline itself for better service in the future in Vietnam.

## 2. Materials and Methods

### 2.1. Study Design and Recruitment Procedure

This was a cross-sectional study, carried out among 1648 callers who sought smoking cessation services via the national counseling quitline (18006606, hosted by the Respiratory Center at Bach Mai Hospital, Hanoi, Vietnam) from September 2015 to May 2016. We offered smoking cessation services, including daily toll-free telephone counseling for nicotine treatment with 10 certified counselors, from 8:00 a.m. to 10:00 p.m. Convenience sampling was applied to recruit 469 callers from 1648 clients of the quitline based on eligibility criteria: (i) being aged from 18 years old; (ii) having used the quitline service by calling and experiencing counseling; and (iii) having consented to take part in a 10 to 15 min telephone interview.

Out of 12,953 calls to the quitline, we excluded 3081 callers who did not set any counseling appointments. Then, we identified 1648 eligible participants from 4148 calls and contacted them again via telephone to introduce the study and invite them to participate in the study. We excluded 1128 participants whom we were unable to contact after trying three times. Finally, a total of 469 participants were recruited from 520 successful callings, achieving a 28.4% response rate.

### 2.2. Measures

The structured questionnaire for the telephone survey was designed to assess demographic characteristics (e.g., gender, age, educational attainment, marriage status, occupation, and living area), history of smoking (e.g., types of tobacco used and frequency of cigarette usage), and quitting patterns before and after using the quitline (e.g., number of quit attempts, duration of smoking abstinence, methods used during quit attempts, and reasons for lapse and relapse). This survey was developed as an important component of the national quitline program, funded by the Ministry of Health in Vietnam. The questionnaire was pre-piloted with five callers and revisions before being applied in the main survey. Some items were culturally adapted via forward and backward translation from the English version (e.g., items related to quitting abstinence).

#### 2.2.1. Quitline Outcomes

Quitline outcomes were measured by collecting data related to smoking abstinence based on the well-known Transtheoretical Model or Stage of Change for smoking cessation developed by Prochaska, DiClemente, and Velicer et al. [19,20]. The term “*process of smoking cessation*” was used to demonstrate five stages of smoking behavior change, including (1) *pre-contemplation*, where a smoker has no intention to quit smoking or intends to quit after 12 months or longer; (2) *contemplation*, where a smoker is aware of health problems due to smoking and intends to quit within six months; (3) *preparation*, where a smoker intends to quit smoking within 1 month; (4) *action*, where the individual stops smoking and maintains early abstinence; and (5) *maintenance*, where the smoker achieves six months of abstinence and maintains efforts. Participants were asked to remember quit attempts and the duration of abstinence in the past 12 months. Prevalence of abstinence was measured based on self-reported assessment and was categorized based on the stage of change and relapse progress. Based on this model, data on abstinence were collected as below:

(i) Contemplation—intention to quit within six months;
(ii) Preparation—intention to quit smoking within 1 month;
(iii) Action—any 24 h quit attempt after telephone counseling and early abstinence (actively stopping for an up to 7 day point PA);
(iv) Maintenance—achieving six months of abstinence and maintaining efforts to resist relapse (from 7 days to 6 months of prolonged PA);
(v) Successful quit attempts—having had a more than 6 month prolonged PA;
(vi) Lapse—any puff during quit attempts [21,22];
(vii) Relapse—smoking at least five cigarettes per day across three consecutive days [23].

We did measure changes in abstinence based on the duration of smoking abstinence before and after using the quitline. Callers who reported more days smoke-free after using quitline compared to prior to using it were referred to as having had “positive change of abstinence” (Y/N) compared to their counterpart peers. Additionally, self-efficacy was measured by the confidence to quit smoking via a Likert-scale of ten different scores (0 meaning not at all confident and 10 totally confident) and the question “How did you feel when trying a quit attempt after the telephone counseling?”. Participants who answered, “easier to quit than before” were referred to as having had a positive change of self-efficacy. This scale was recommended by the Mayo Clinic for measuring confidence in quitting smoking [24].

#### 2.2.2. Quitline Uptake

Participants were assessed on quitline uptake based on the questions “How many times have you contacted the quitline operators?” and “Have you ever actively tried to recontact the quitline after the first instance of counseling?”.

#### 2.2.3. Associated Factors

Satisfaction for service quality was measured using the Likert-scale of ten different scores (0meaning not at all satisfied and 10 totally satisfied). Participants also collected data regarding their history of smoking cessation; additional methods used for smoking cessation, e.g., NRT; any support from family or friends; reasons for relapse; intentions to quit in the future; and expectations for better services.

### 2.3. Statistical Analysis

The primary endpoints were (i) 7 day point PA, (ii) 6 month prolonged PA, (iii) changes in abstinence duration pre- and post-quitline, and (iv) self-reported quitline recontact.

Statistical analysis was performed using STATA version 12.0 (Stata Corp. LP, College Station, TX, USA). For descriptive statistics of categorical variables, the Chi-square test was carried out to detect any significant difference in sampling characteristics (e.g., socio-economic factors, history of being smoke-free, quit attempts, and abstinence stage of changes) by current smokers and non-smokers via a comparison of proportions using odds ratios (OR). For continuous variables, a two sample t-test was used to compare age (mean and SD) between smokers and non-smokers. For some variables that were not found to be normally distributed (e.g., score of satisfaction, confidence to quit, and number of abstinence days), nonparametric statistics were used (i.e., median, inter-quartile range (IQR), and the Wilcoxon Mann-Whitney test for comparisons).

To understand the pattern of abstinence progress and to determine how telephone counseling can contribute to improving smoking abstinence among quitters, we ran an analysis for paired data to compare the abstinence differences before and after using quitline. We ran McNemar’s test and the Wilcoxon signed-rank test to detect changes in PA for dichotomous variables and the median of abstinence days, respectively.

Finally, to control for any existing confounders, multivariate logistic regression was performed to determine any factors associated with the likelihood of a positive change of abstinence duration pre- and post-quitline, successful quit attempts (6 month prolonged PA), and the possibility of recontacting quitline. The stepwise backward approach was employed with imputation of all variables that were significantly associated with outcomes of interest in the Chi-square and Mann-Whitney U-tests, including demographic characteristics (age, marriage status, educational attainment, and occupation), history of smoking abstinence (methods used to quit, number of quit attempts, and confidence to quit smoking), and service usage (quitline uptake, service satisfaction, and having had any supporters during quit attempts). A *p*-value of log-likelihood of 0.2 as the cut-off point was applied for all variables to be included in the final models. Significance levels were set at the level of *p*-value ≤ 0.05.

### 2.4. Ethics Approval

Ethics approval was obtained from the Institutional Review Board of Bach Mai Hospital (Ethics approval number: 10/QD—VNRS, dated 2 February 2018). All callers were provided the purpose, potential risks, and benefits of the survey, and oral consent was obtained before the interview.

## 3. Results

Table 1 shows the sample characteristics by smoking status at the time of the survey. Among the total 469 male clients of the quitline service, most were married (82.5%), had achieved high school accomplishment (51.2%), and worked as blue-collar employees (40.7%), and roughly half resided in rural areas (53.7%). The mean age was 39.7 (SD: 13.1), and no significant difference in mean age between smokers and non-smokers was found (*p* = 0.07). We observed that there was no significant difference in mean age and demographic background (e.g., in education, occupation, living area, or marriage status) between smokers and non-smokers at the time of survey (*p* > 0.05).

Table 2 indicates tobacco usage patterns prior to and after using quitline services among 321 current smokers (68.4%) and 148 quitters (31.6%) who had been abstinent. Before using the quitline, most of the participants aimed to contact the quitline to find quit help (73.4%) because they had experienced quit attempts before (75.3%) and half of the participants had experienced more than 7 days of smoking abstinence (49.8%). However, many participants had relapsed in their last quit attempt (73.6%). The most common method used in the last quit attempt was self-help (65.4%), and a few smokers had been using nicotine replacement therapy in conjunction (6%). Quitline uptake was low in the last 12 months because most of the participants did not redial the operator (41.6%) and roughly half of the smokers engaged support from family or friends during the quit period (59.9%).

After using the quitline, most of the clients were satisfied with the quality of service (88.5%), felt more confident about quitting (74.3%), and took early action via the first quit attempt (81.7%). However, only 23.9% of clients maintained more than 7 days of abstinence, and 5.1% were considered quitters (had a more than 6 month prolonged period of abstinence). When comparing the difference in abstinence duration (in days) before and after using quitline, it can be observed that 24.9% of participants positively increased the duration of their abstinence episode, but for more than half of the smokers, this remained unchanged (69.5%). The main reason for smoking relapses during quit attempts was exposure to peers in smoke environments (51.6%). The favorite additional supports in conjunction with quitline were redirecting counseling from the operator (75.9%) and SMS reminders (67.8%). The callers who were currently non-smoking at the time of the survey had a significantly higher mean score of self-efficacy (8.8/10, SD = 1.4 versus 6.7, SD = 2.2), mean score of service satisfaction (8.7/10, SD = 1.3), and mean day of smoking abstinence immediately after using the quitline (10.2, SD = 28) compared to their peer smokers, respectively (smokers had a mean score of self-efficacy of 6.7, SD = 2.2; a mean score of satisfaction of 7.6, SD = 1.8; and a mean day of smoking abstinence of 2.8, SD = 8.4).

Table 3 reports the changes in point abstinence prevalence pre- versus post-quitline use among 148 quitters. It was observed that smokers’ movement toward prolonged abstinence (i.e., being in the maintaining stopping stage) changed significantly over the twelve months of quitline utilization (*p* < 0.001). The odds ratio from matched data shows that after using quitline services, smokers were more likely to reach longer abstinence (i.e., a more than 7 day abstinence period) compared to prior to using quitline. In addition, after receiving quitline services, participants were less likely to relapse (OR = 0.16).

Table 4 reports the factors related to quitline outcomes via three logistic models (i.e., positive change in smoking abstinence, successful quitters or more than a 6 month abstinence period, and recontacting an operator in the past 12 months). The data shows that the likelihood of significantly increasing smoking abstinence was higher among smokers who used NRT (OR 5.7, 95% CI 3.17–10.11), who had a higher satisfaction score for quitline quality (OR 1.5, 95% CI 1.23–1.82), who had had a pre-existing quit attempt prior to using quitline, and who were jobless (i.e., were students, retired persons, or did housework), compared to the counterpart groups. However, those smokers who also used NRT were five times more likely to maintain abstinence and successfully stop smoking (OR 5.0, 95% CI 2.0–12.8). In addition, a certain trend toward significance (*p* = 0.09) was seen in that smokers who had experienced pre-existing quit attempts were more likely to be a successful quitter (OR 2.3, 95% CI (0.9–6.2). Clients were more likely to redial the quitline if they were offered NRT and were actively being re-contacted by the operator (OR 2.4, 95% CI 1.1–5.1).

## 4. Discussion

In light of Vietnam being within the top 15 countries of high prevalence of smoking among male adults [25], the smoking cessation Quitline in Vietnam is expected to contribute to current efforts toward reducing smoking-related mortality and morbidity and reaching the national target, by 2020, of reducing the smoking prevalence among male adults from 47.4% (2011) to 39% (2020) [10]. Launched in 2015, the telephone counseling center (number 18006606) is the first national toll-free Quitline service to provide timely support for smokers who are struggling with nicotine dependence and craving symptoms during a quit attempt. The purpose of this study was to examine the impact of national telephone counselling in improving smoking abstinence outcomes and identify factors that are associated with a change in smoking behaviors based on data from cross-sectional data in Vietnam. The most prominent finding to emerge from the analysis is that although the quit rate (judged by a 6 month period of prolonged abstinence) of callers using the quitline was 5.1%, which is slightly lower than other telephone counseling interventions in other countries (ranging from 7% to 10%) [18], this service helped callers become more motivated to quit, with 80% of participants trying a quit attempt after counseling and one-fifth of participants moving to a maintaining stage (having up to 6 months prolonged abstinence). This finding broadly supports the work of other studies in this area linking telephone support with improving motivation to quit smoking (risk ratio 1.38, 95% CI 1.19–1.61), based on results from a systematic review of 14 trials [18]. This result also reflects the potential benefits and efficacy of a proactive quitline in identifying and screening smokers who interested in quitting, providing counseling, and referring these individuals to further community support [26].

Among non-smokers at the time of survey who were attempting to quit, we observed a positive change in smoking behaviors. After using Quitline, the callers, who had experienced quit attempts and relapses in the past, tended to have a positive shift toward the next stage of change with prolonged abstinence. In other words, they were more likely to maintain abstinence and move toward successfully stopping by resisting smoking temptations and strengthening self-confidence and motivation to quit. This result is consistent with literature which confirms that counseling and support from a quitline are associated with improving quitting outcomes and increasing self-efficacy [27]. However, these results do not support a causal relationship and future cohort studies, or experimental trials, are necessary to confirm the actual efficacy of a quitline that changes over time. In addition, a further study with more focus on how quitline utilization can contribute to improving well-being and quality of life and reduce daily stressors among callers is recommended because evidence shows that when smokers try to give up smoking habits, there is an observed better quality of life [28].

One critical question is how to maintain quitline uptake. We found that client satisfaction and being actively recontacted by the operator are two crucial factors associated with increased quitline use. This also accords with earlier observations which have shown that callers who receive multiple instances of call-back counseling [29] and satisfaction scores are associated with service uptake and greater improvement of long-term abstinence and success rates [30]. To improve patient satisfaction, the quitline should offer three to five instances of call-back counselling [18] in combination with how to enhance the skills of counselors because counselor effects contribute to increasing quit rates by 2% [31]. In addition, this telephone counseling might consider taking advantage of eHealth/mHealth innovations in order to provide further web-based or text messaging tracking tools since a strong relationship between the application of eHealth and/or mHealth programs, and greater improvement of prolonged abstinence has been reported in the literature [32]. Furthermore, it is necessary to deliver and facilitate information about quitline services via nurse or doctor referral in the health care system, as this has been proven effective in promoting awareness [33] and uptake of quitlines in public health practice, especially among chronic patients (e.g., those with chronic obstructive pulmonary disease, heart disease, or lung disease) who need to cease smoking during their treatment [34]. Further studies have confirmed that telephone counseling is associated with higher effectiveness when combined with brief quit advice from healthcare staff [18].

The most important clinically relevant finding was that the positive change in smoking abstinence days and successful quitters was strongly significantly associated with the used of NRT. Consistent with the literature, this research found that participants who reported using NRT in conjunction with quitline counseling were more likely to reach 6 month prolonged abstinence than non-NRT callers. An implication of this is the possibility of offering NRT in combination with the national quitline to increase the quitline uptake and improve prolonged abstinence because this approach might help to increase 6 month PA by 10% (for 2 weeks) and 13% (for 6 weeks) [35]. For short-term outcomes, other studies have also found that NRT utilization with phone counseling can improve the achievement of 7 day point PA from 10.3% (before being offered NRT) to 14.9% (after being offered NRT) [36]. Further research should be undertaken to investigate the feasibility and cost-effectiveness of this strategy and whether callers can afford the service costs and their willingness to pay if NRT is unable to be offered free of charge.

The most important clinically relevant finding was the positive change in abstinence among quitters before and after using the quitline. We observed that after using quitline services, callers who had experienced early abstinence were more likely to shift toward the next step of the stage of change (i.e., prolonged abstinence or 6 month prolonged abstinence) compared to prior to using quitline. This result may be explained by the fact that for callers who have already had adequately pre-existing quit attempts and self-efficacy to quit but have still failed or relapsed for various reasons (e.g., craving symptoms or peer temptations), quitline counselors can supply useful and tailored tips or quit advice that helps these individuals to resist slips or lapses and successfully maintain prolonged abstinence [22]. In accordance with the present results, previous studies have demonstrated that pre-existing quitting attempts are associated with the confidence to stop and to maintain a smoke cessation plan [17]. Thus, in the future, quitline counseling should build up a client profile and offer tailored and personalized counseling based on callers’ quitting characteristics rather than providing intensive counseling for all callers [18].

The present study has been one of the first attempts to thoroughly examine the early impacts of Vietnam’s unique national telephone counseling service for smoking cessation since its initiation in 2015. The findings of this research provide insights into the premilitary understanding of how quitline services can better improve quality and quit outcomes. However, some drawbacks should be acknowledged. Firstly, one source of weakness in this study which could have affected the measurements of smoking abstinence outcomes was the use of self-reported data without biochemical verification methods (e.g., breath carbon monoxide testing or cotinine urinalysis to validate the self-reported assessment of smoking abstinence). In addition, recall bias within 12 months might result in detection bias that overestimates or underestimates the quitline outcomes. Secondly, using cross-sectional data, this study was unable to assess how quit rates can change over time and how this relates to quitline uptake. Thirdly, in observational studies, there is the potential for bias from sample recruitment due to the use of a single quitline center that might not be fully representative of the general population of Vietnamese smokers. In addition, all of the participants in this study were male due to the low preference for smoking among women (1.1%), yet there is a possible stigma against women accessing smoking cessation services or Asian cultural norms preventing women from accessing smoking cessation services [37]. Despite the gender disparity in the quitline trajectory, the findings here reflected the current male dominated tobacco use and men being the main target clients of quitline services, which is necessary to consider when designing quitting messages or content in the future. Ultimately, these findings cannot be extrapolated to all smokers due to the participants being mainly from the Northern area.

## 5. Conclusions

The present study appears to have been the first study to examine the impact of the national smoking cessation Quitline in Vietnam. One of the more significant findings to emerge from this study is that the callers were more likely to improve quit attempts and feel confident about quitting after using Quitline. The two major clinical implications of this study are the important role of quitline satisfaction and the future consideration of offering NRT in conjunction with quitline, which was significantly associated with greater improvement of prolonged abstinence among male Vietnamese smokers.

## Figures and Tables

**Table 1:** Sample characteristics by smoking status (*n* = 469).

Characteristics	Currently Smoking		Total	*p*-Value
	Yes	No		
	*n* (%)	*n* (%)	*n* (%)	
**Gender (*n* = 469)**				
Male	321 (100)	148 (100)	469 (100)	0.001
Female	0	0	0	
**Marital status (*n* = 469)**				
Unmarried	59 (18.4)	23 (15.5)	82 (17.5)	0.45
Married or de facto husband/wife	262 (81.6)	125 (84.5)	387 (82.5)	
**Education attainment (*n* = 469)**				
Below high school	97 (30.2)	46 (31.1)	143 30.5)	0.83
High school	167 (52)	73 (49.3)	240 (51.2)	
Above high school	57 (17.8)	29 (19.6)	86 (18.3)	
**Occupation (*n* = 469)**				
Blue-collar workers	134 (41.7)	57 (38.5)	191 (40.7)	0.59
White-collar workers	86 (26.8)	41 (27.7)	127 (27.1)	
No job (students, housework)	65 (20.3)	37 (25)	102 (21.7)	
Others	36 (11.2)	13 (8.8)	49 (10.5)	
**Living area (*n* = 469)**				
Rural	169 (52,7)	83 (56.1)	252 (53.7)	0.49
Urban	152 (47.4)	65 (43.9)	217 (46.3)	

**Table 2:** Smoking patterns by smoking status (*n* = 469).

Characteristics	Smokers (*n* = 321)	Quitters (*n* = 148)	Total	*p*-Value
	*n* (%)	*n* (%)	*n* (%)	
**Prior to using the quitline**				
**Reason for calling the quitline (*n* = 469)**				
Seeking advice for quit help	234 (72.9)	113 (76.4)	347 (73.4)	0.43
Seeking more information on smoking cessation	154 (47.9)	68 (45.9)	222 (47.3)	0.68
Seeking how to maintain quit attempts and stopping	24 (7.4)	19 (12.8)	43 (9.2)	0.06
History of quit attempts (yes versus no)	256 (79.7)	97 (65.5)	353 (75.3)	0.001
**Stage of change (*n* = 446)**				
Contemplation/preparation	65 (21.4)	51 (35.9)	116 (26.1)	0.013
History of up to 7 day point prevalence abstinence (PA)	80 (26.3)	28 (19.7)	108 (24.2)	
History of 7 day point PA to 6 month point PA	129 (42.4)	51 (35.9)	180 (40.4)	
History of more than 6 months prolonged PA	30 (9.9)	12 (8.5)	42 (9.4)	
**Relapse in the last quit attempt (yes versus no) (*n* = 460)**	195 (76.2)	65 (67)	260 (73.6)	0.081
**Other methods used for support during the last quit attempt (*n* = 353)**				
Nicotine replacement therapy (NRT) (Bupropion, Varenicline)	14 (5.5)	7 (7.2)	21 (5.9)	0.001
Self-help	225 (87.9)	6 (6.2)	231 (65.4)	
Quitline	3 (1.2)	2 (2.1)	5 (1.4)	
Switching to smokeless tobacco	14 (5.5)	82 (84.5)	96 (27.2)	
**History of using quitline within 12 months (*n* = 457)**				
No calling	124 (39.5)	66 (46.2)	190 (41.6)	0.553
One time	96 (30.6)	40 (27.9)	136 (29.8)	
Two times	53 (16.9)	19 (13.3)	72 (15.7)	
Three times or more	41 (13.1)	18 (12.6)	59 (12.9)	
**Having supporters (friends or family) during quit attempt (*n* = 461)**	172 (53.6)	109 (73.6)	281 (59.9)	0.001
**After using quitline**				
**Satisfaction with quitline service (yes versus no) (*n* = 469)**	271 (84.4)	144 (97.3)	415 (88.5)	0.001
**Feeling more confident about quitting (yes versus no) (*n* = 462)**	209 (65.5)	138 (93.2)	347 (74.3)	0.001
**Any quit attempts after counselling (*n* = 464)**	237 (74.5)	143 (97.3)	380 (81.7)	0.001
**Stage of change after using the quitline (*n* = 469)**				
Current smokers	321 (100)	0	321 (68.4)	0.001
Up to 7 day point PA	0	12 (8.1)	12 (2.6)	
From 7 day point PA to 6 month point PA	0	112 (75.7)	112 (23.9)	
More than 6 month prolonged PA	0	24 (16.2)	24 (5.1)	
**Difference in abstinence duration pre- and post-quitline (*n* = 469)**				
Increased duration of smoking abstinence	0 (0)	117 (79.1)	117 (24.9)	0.001
Unchanged	317 (98.7)	9 (6.1)	326 (69.5)	
Decreased duration of smoking abstinence	4 (1.3)	22 (14.9)	26 (5.5)	
**Reason for relapse after using the quitline (*n* = 469)**				
Experienced craving symptoms	133 (54.9)	34 (24.8)	167 (44.1)	0.001
Exposed to peers or smoke environment	154 (63.9)	41 (29.9)	195 (51.6)	0.001
Experienced stress	108 (44.8)	16 (11.7)	124 (32.8)	0.001
**Favorite additional supports (*n* = 469)**				
Redirecting counselling from operator	262 (81.6)	94 (63.5)	356 (75.9)	0.001
SMS reminders	229 (71.3)	89 (60.1)	318 (67.8)	0.016
Face to face counselling	222 (69.2)	74 (50)	296 (63.1)	0.001
Peer supports	208 (64.8)	78 (52.7)	286 (60.9)	0.013
NRT	250 (77.9)	54 (36.5)	304 (64.8)	0.001

**Table 3:** Outcome of smoking abstinence pre- and post-quitline use among quitters (*n* = 148). Legend: OR, odds ratio.

Post-Quitline Abstinence	Pre-Quitline Abstinence				
	Yes	No	Total	*p*-Value	OR
Early abstinence (≤7 days) (yes versus no)	2 (7.1)	26 (7.5)	28 (7.4)	0.006	0.3
Maintained (7 days to 6 months) (yes versus no)	39 (76.5)	12 (75.3)	51 (75.7)	0.001	6.08
Stopped (≥6 months) (yes versus no)	6 (50)	18 (13.2)	24 (16.2)	0.02	3
Relapsed during last quit attempt period (yes versus no)	28 (24.6)	14 (43.7)	42 (28.8)	0.001	0.16
	Median (range)	Median (range)	Median (range)		
Median of abstinence days	90 (0–720)	7 (0–1080)	60 (0–720)	0.001	

**Table 4:** Factors associated with the quitline outcomes.

Characteristics	Model 1: Positive Change in Smoking Abstinence Days (*n* = 465)	Model 2: Being a Successful Quitter within 12 Months (*n* = 305)	Model 3: Recontacting the Operator within 12 Months (*n* = 465)
	OR (95% CI)	OR (95% CI)	OR (95% CI)
Having used NRT (yes versus no)	5.7 (3.17–10.11) ***	5.0 (2.0–12.8) ***	0.3 (0.2–0.5) ***
Service satisfaction score	1.5 (1.23–1.82) ***		
Pre-existing quit attempt before using the quitline (yes versus no)	0.2 (0.05–0.48) ***	2.3 (0.9–6.2) *	
Feeling confident about quitting (yes verus no)	0.3 (0.10–0.73) **		0.6 (0.4–1.0) *
Occupation (no job versus employment)	2.3 (1.23–4.18) ***		0.6 (0.3–1.3)
Living area (urban versus rural)	0.6 (0.38–1.07) *		
Age group (>59 years versus <59 years)	0.3 (0.09–1.08) *		
Marriage status (married versus unmarried)	1.9 (0.93–3.84) *		
Participated in smoking cessation course (yes versus no)	0.7 (0.38–1.20)		
Education level (above high school versus below high school)		0.2 (0.0–0.9) **	
Age group (45-59 years versus >59 years)		2.0 (0.8–4.7)	
Having been recontacted by the operator (yes versus no)			2.4 (1.1–5.1) **
SMS reminder (yes versus no)			0.5 (0.3–1.0) *
Being supported by friends or family during the quit attempt (yes versus no)			0.7 (0.5–1.1)

*** *p* < 0.01, ** *p* < 0.05, * *p* < 0.1.
